# Supplementary material for: Summon a demon and bind it: A grounded theory of LLM red teaming
Source: PLoS One. 2025 Jan 15;20(1):e0314658. doi: 10.1371/journal.pone.0314658 (PMC11734899; doi:10.1371/journal.pone.0314658)
Supplement: S1 Appendix — (ZIP) [file pone.0314658.s001.zip › Appendix A.pdf]

A Interview guide

A.1 Intro to the interview

- Intro of the research
- Interviewer name
- Interviewer affiliation

Inform about context: We're looking at how people generate undesirable language model outputs

Is it OK for us to record this & use the recording in research?

(press record)

(is it recording?)

Confirm it's OK that we record.

A.2 Demographics

Just a few broad demographic questions to help us know how broad our data is

- Would you like to tell us the first digit of your age, i.e. decade?
- Would you like to tell us your occupation / job title?
- Would you like to tell us your gender, however you describe that?
- Do you have a highest level of education that you'd like to share?

A.3 Main questions

- How would you refer to <X, this activity you've done, whatever the interviewee group calls it>, with language models?
  - Are there any other terms for this? That you prefer?
  - How do you define the activity?
- Is there an example of how you did X in practice?
- Why, what was the purpose of doing it?
- ** What are/were you looking for when you X?
  - What does a success look like / what's an interesting result?
- ** What's your process like?
- Where do you go, to interact with the model? (e.g. OpenAI playground, API, deployment context, ...)
- Can you describe in as much detail as possible what you are thinking about when you design an input?

  - Imagine you have a toolbox. What tools are in there?
  - Maybe none of the tools work. What intuitions might you use?
- What do you do if an input doesn't work out?
- ** So imagine you want to make a model <perform example misbehaviour>. Can we try that? Can you show me? – x2-3
  - *[cook meth; advocate for nazism; be sexist; leak a personal address; give misinformation]*
  - Can you talk me through what you're thinking?
  - What output are you hoping for?
  - What do you think of that output?
  - What's going on in your prompt?
- What do you do *besides* editing the text?
- How do you record your results?
- Is this an activity that there's a strategy for?
- How long do you spend on it?
- What do you do with the outputs?
  - Do you track your progress?
- Are there any **external** motivating factors? (maybe just a cool story)

A.3.1 Outro/debriefing questions

- SLOW QUESTION How do you think other people perceive this activity?
- SLOW QUESTION Now we've spoken, do you have any thoughts about what effect this kind of knowledge might have on others?
- Do you have any questions for me/us?
- Is there anything else we didn't cover, that you'd like to add?
- Is there anybody else you think could be interesting for us to talk to?
- When we publish, would you like to be mentioned in the acknowledgments? (under what name?)
